# Combination of handgrip strength and high-sensitivity modified Glasgow prognostic score predicts survival outcomes in patients with colon cancer

**DOI:** 10.3389/fnut.2024.1421560

**Published:** 2024-07-01

**Authors:** Yifan Shi, Yuting Sun, Xiaoming Shen, Zenghui Yang, Binghua Xu, Chuanqing Bao

**Affiliations:** ^1^Department of Gastrointestinal Surgery, Affiliated Hospital of Jiangnan University, Wuxi, China; ^2^Department of General Surgery, Jiangnan University Medical Center, Wuxi, China

**Keywords:** handgrip strength, HS-mGPS, colon cancer, postoperative survival, nomogram

## Abstract

**Objective:**

Handgrip strength (HGS) and the high-sensitivity modified Glasgow prognostic score (HS-mGPS) are associated with the survival of patients with cancer. However, no studies have investigated the combined effect of HGS and HS-mGPS on the overall survival (OS) of patients with colon cancer.

**Methods:**

Prospective follow-up data of colon cancer patients undergoing radical resection from April, 2016 to September, 2019 were retrospectively collected. We combined the HGS and HS-mGPS to create a new composite index, HGS-HS-mGPS. The hazard ratio (HR) and 95% confidence interval (CI) were calculated using Cox regression models to assess the association between variables and OS. Risk factors on OS rates were investigated by Cox analyses and the nomogram was constructed using significant predictors and HGS-HS-mGPS. The predictive performance of the nomogram was evaluated by receiver operating characteristic curve and calibration curve.

**Results:**

This study included a total of 811 patients, of which 446 (55.0%) were male. The HGS optimal cut-off values of male and female patients were 28.8 and 19.72 kg, respectively. Multivariate analysis revealed that low HGS and high HS-mGPS were independent risk factors of colon cancer after adjusting confounders (adjusted HR = 3.20; 95% CI: 2.27–4.50; *p* < 0.001 and adjusted HR = 1.55; 95% CI: 1.12–2.14; *p* = 0.008 respectively). Patients with low HGS and high HS-mGPS had a 10.76-fold higher mortality risk than those with neither (adjusted HR = 10.76; 95% CI: 5.38–21.54; *p* < 0.001). A nomogram predicting 1-, 3-, and 5 year OS was constructed based on three clinicopathologic prognostic factors. Importantly, incorporating HGS-HS-mGPS into the nomogram model meaningfully improved the predictive performance. The decision curve analyses demonstrated the application value of the HGS-HS-mGPS nomogram for predicting OS of patients with colon cancer.

**Conclusion:**

HGS-HS-mGPS is associated with the survival of patients with colon cancer. These findings indicate the usefulness of HGS and HS-mGPS measurements in clinical practice for improving patient assessment, cancer prognosis, and precise intervention.

## 1 Introduction

Colon cancer is the fourth most common cancer worldwide with approximately 1.1 million diagnoses annually (1). In 2020, the global mortality rate for colon cancer was approximately 5.8 per 100,000 individuals (2). Patients with colon cancer are susceptible to malnutrition, ranging from 30 to 50%, because of the impaired intake, digestion, and absorption of nutrients (3, 4). The poor nutritional status associated with colon cancer significantly influences patient’s clinical outcomes, including increased postoperative complications, prolonged hospital stays, and elevated cancer mortality (5, 6). It is well-accepted that malnutrition indirectly accounts for at least 20% of deaths among all cancer patients (7). Therefore, it is crucial to identify nutritional indicators that are easy to measure in clinical practice for predicting the prognosis of colon cancer patients.

Skeletal muscle mass (SMM) is a common nutritional indicator, reflecting protein-energy malnutrition (8). However, evaluating SMM requires computed tomography, which is costly and places a significant financial burden on patients (9). Handgrip strength (HGS) is considered a reliable and cost-effective method for predicting muscle mass (10). A previous study indicated that HGS is a predictor of malnutrition in hospitalized patients with cancer (11). Low HGS was inversely associated with the survival outcomes of site-specific cancers (lung and breast), cardiovascular, and respiratory diseases, but this differed in male and female patients (12, 13). In addition, a multicenter observational study demonstrated a close association between low HGS and cancer mortality (14). Hence, we speculated that HGS could reflect skeletal muscle mass and exert a predictive role in the prognosis of colon cancer.

Additionally, systemic inflammation is a crucial driving force of skeletal muscle mass loss in patients with cancer-associated cachexia, influencing cancer progression and prognosis (15). The high-sensitivity modified Glasgow prognostic score (HS-mGPS) that combines C-reactive protein (CRP) and albumin levels reflects the inflammation and nutritional status of the patients. Previous investigations indicated that the HS-mGPS had prognostic value in patients with lung, gastric, and esophageal cancers, independent of the tumor stage (16). Compared with the conventional mGPS, the HS-mGPS has been demonstrated to be a superior prognostic factor for therapeutic response and long-term survival in various malignancies (17, 18). In addition, HS-mGPS may also be an independent prognostic indicator for advanced colon cancer (19).

Although the prognostic values of HGS and HS-mGPS have been previously reported, no studies have investigated whether combining HGS and HS-mGPS (HGS-HS-mGPS) is associated with the prognosis of colon cancer. Therefore, this study aimed to probe the sex-specific cut-off values of HGS and examine the associations of HGS, HS-mGPS, and HGS-HS-mGPS with postoperative overall survival (OS) in patients with colon cancer.

## 2 Methods

### 2.1 Patient selection

Prospective follow-up data of patients with colon cancer treated at the Affiliated Hospital of Jiangnan University from April 2016 to September 2019 were retrospectively collected. The inclusion criteria were as follows: (1) age ≥ 18 years old, (2) postoperative pathological diagnosis of colon adenocarcinoma, (3) receiving radical resection, and (4) TNM stage I to III. The exclusion criteria included: (1) patients with incomplete clinical data, (2) death in the hospital or within 30 days after surgery, (3) preoperative neoadjuvant therapy, (4) concurrent secondary tumor or rectal cancer, (5) previous history of other malignancies, and (6) pregnancy. This study was approved by the ethics committee of the Affiliated Hospital of Jiangnan University (No. 20230046) and conducted following the Declaration of Helsinki.

### 2.2 Data collection

Through the prospectively maintained colon cancer database, the following information was carefully collected: demographics and clinical features [sex, age, body mass index (BMI), HGS, age-adjusted Charlson Comorbidity Index (aCCI), carcinoembryonic antigen (CEA), albumin, CRP, and bowel obstruction], surgical characteristics [tumor location, operation type and postoperative comprehensive complication index (CCI)], pathological information (tumor differentiation and stage), and postoperative chemotherapy. These variables of interest were derived from clinical experience and previous studies. Postoperative complications were evaluated using the CCI scores^1^, which integrates all postoperative complications with their corresponding severities, on a scale ranging from 0 (no complications) to 100 (death) (20). A CCI score of ≥26.2 considered as the threshold for serious complications (21). The tumor staging was determined based on pathological reports using the American Joint Committee on Cancer (AJCC) 8th edition.

### 2.3 Definition of variables

#### 2.3.1 HGS

HGS was measured using an electronic dynamometer (EH101, CAMRY, Guangdong, China). The handle was individually adjusted according to the size of the patient’s hand. During the measurement, the patients sat straight with the arms resting on the armrest and the elbows bending at 90°. Patients were instructed to grip the handle with maximal force during 3 s for three consecutive times and the maximal HGS was taken (10). The patients were divided into low and high HGS groups according to sex-specific cut-off values.

#### 2.3.2 HS-mGPS

The HS-mGPS was determined as follows: patients with both increased CRP levels (>3 mg/L) and hypoalbuminemia (<35 g/L), with one of these variables, and without these variables were allocated scores of 2, 1, and 0, respectively (16). A HS-mGPS score of 0 was defined as low HS-mGPS, while scores higher than 0 were determined as high HS-mGPS.

#### 2.3.3 HGS-HS-mGPS

For combining HGS and HS-mGPS, the low or high HGS and low or high HS-mGPS were cross-classified into four categories. Patients with both low HGS and high HS-mGPS, only low HGS, only high HS-mGPS, and neither were defined as the high risk, median risk 1, median risk 2, and low risk groups, respectively.

### 2.4 Study outcome

The primary outcome of this study was OS, defined as the time from the diagnosis of colon cancer to the last follow-up or death from any cause. The living status and treatment information were obtained through telephone consultations, outpatient, or inpatient follow-ups after completing the primary therapy. Follow-up visits were conducted every three months for 2 years postoperatively and every 6 months thereafter. The end date of the follow-up was March 31, 2023.

### 2.5 Statistical analysis

Statistical analysis was performed by the R software (version 4.0.3; http://www.r-project.org/). Continuous variables included in this study were presented as the means and standard deviations (SD), while categorical variables were presented as frequencies and corresponding percentages. Baseline characteristics between the two groups were compared using independent Student’s *t*-tests for continuous variables and Chi-squared tests for categorical variables.

The sex-specific cut-off points of HGS were calculated with the package of survminer. To assess the association between variables and OS, we calculated the hazard ratio (HR) and 95% confidence interval (CI). In the multivariate Cox regression models, model 1 was unadjusted, model 2 was adjusted for sex, age, BMI, aCCI, tumor T and N stage, and model 3 was adjusted for sex, age, BMI, aCCI, CEA, bowel obstruction, tumor location, operation type, CCI ≥ 26.2, tumor differentiation, T stage, N stage, and postoperative chemotherapy. Survival curves were delineated by the Kaplan–Meier analysis according to different prognostic risks and compared using the log-rank test. The nomogram was constructed based on significant predictors using R version 4.0.3. Five-fold cross-validation was performed 1,000 times for internal validation of the model. The predictive performance of the nomogram was evaluated using the concordance index (C-index), area under the curve (AUC), and calibration curve. Statistical significance was set at two-sided *p* < 0.05.

## 3 Results

### 3.1 Basic characteristics

**Figure 1:**
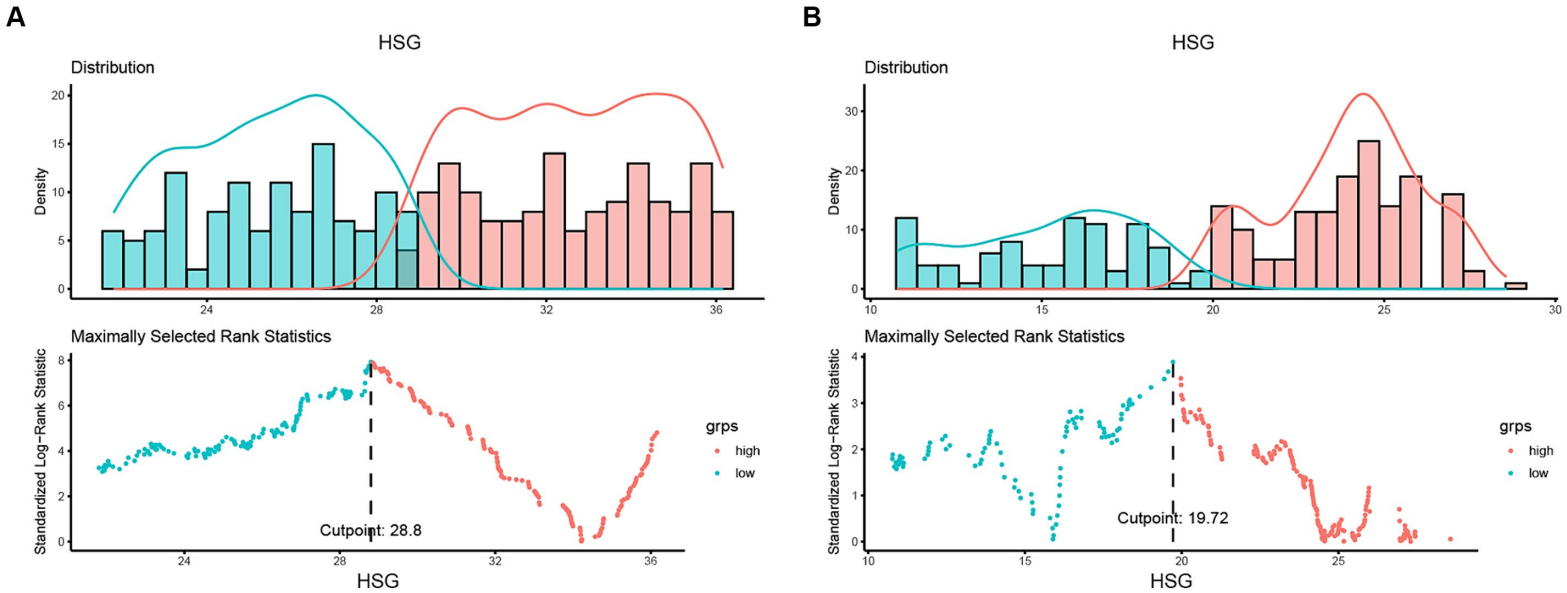
Determining cut-off values of HGS based on sex-specific strata. The optimal HGS cut-off values in patients with colon cancer were <28.8 kg for males **(A)** and <19.72 kg for females **(B)**. HGS, handgrip strength.

A total of 993 patients were eligible for inclusion criteria, of whom 135 were excluded and 47 were lost to follow-up (Supplementary Figure S1). Finally, this study included 811 patients with 446 males (55.0%) (Table 1). Among these patients with colon cancer, the optimal cut-off values of HGS were <28.8 kg for males and <19.72 kg for females (Figure 1). The comparison of the baseline characteristics of patients as categorized by their HGS and HS-mGPS levels is presented in Supplementary Table S1. The results showed that low HGS was significantly related to increased CRP level (*p* < 0.001). Both HGS and HS-mGPS were associated with tumor stage (*p* < 0.001 and *p* = 0.008, respectively).

**Table 1:** Baseline characteristics of patients with colon cancer.

Variables	Patients (*n* = 811)
Sex, *n* (%)	
Male	446 (55.0)
Female	365 (45.0)
Age (years), mean (SD)	63.77 (10.1)
Age, *n* (%)	
<60 years	337 (41.6)
≥60 years	474 (58.4)
BMI (kg/m^2^), *n* (%)	
<18.5	63 (7.8)
18.5–24	627 (77.3)
>24	121 (14.9)
HGS (kg), mean (SD)	25.67 (7.55)
aCCI, *n* (%)	
0–1	172 (21.2)
2–3	318 (39.2)
≥4	321 (39.6)
CEA (μg/L), *n* (%)	
<5	492 (60.7)
≥5	319 (39.3)
Albumin (g/L), mean (SD)	38.16 (7.2)
CRP (mg/L), median (interquartile range)	1.92 (1.00,10.21)
HS-mGPS, *n* (%)	
0	413 (50.9)
1	174 (21.5)
2	224 (27.6)
Bowel obstruction, *n* (%)	
No	585 (72.1)
Yes	226 (27.9)
Tumor location, *n* (%)	
Right-sided	311 (38.3)
Transverse	61 (7.5)
Left-sided	439 (54.1)
Operation type, *n* (%)	
Laparoscopy	661 (81.8)
Laparotomy	150 (18.2)
CCI ≥ 26.2, *n* (%)	
No	660 (81.4)
Yes	151 (18.6)
Differentiation, *n* (%)	
High	63 (7.8)
Moderate	345 (42.5)
Low	403 (49.7)
T stage, *n* (%)	
T1	141 (17.4)
T2	186 (22.9)
T3	190 (23.4)
T4	294 (36.3)
N stage, *n* (%)	
N0	453 (55.9)
N1	235 (29.0)
N2	123 (15.2)
TNM stage, *n* (%)	
I	211 (26.0)
II	250 (30.8)
III	350 (43.2)
Postoperative chemotherapy, *n* (%)	
No	371 (45.7)
Yes	440 (54.3)

Continuous variables were presented as means (standard deviations, SD) or median (interquartile range). Categorical variables were expressed as frequencies (percentages). BMI, body mass index; HGS, hand grip strength; aCCI, age-adjusted Charlson comorbidity index; CEA, carcinoembryonic antigen; CRP, C-reactive protein; HS-mGPS, high-sensitivity modified Glasgow prognostic score; CCI, comprehensive complication index.

### 3.2 The associations of HGS, HS-mGPS, and HGS-HS-mGPS with OS

Cox regression analyses adjusted by potential confounders were performed to assess the relationships between each factor and OS in patients with colon cancer and the results are summarized in Table 2. Death risk in low HGS group was 3.2-fold compared to that in the high HGS group (adjusted HR = 3.20; 95% CI: 2.27–4.50; *p* < 0.001). However, continuous HGS did not significantly affect OS (adjusted HR = 0.98; 95% CI: 0.96–1.00; *p* = 0.097). There was a 55% higher risk of death in the high HS-mGPS group than in the low HS-mGPS group (adjusted HR = 1.55; 95% CI: 1.12–2.14; *p* = 0.008). Notably, colon cancer patients with high risk (both low HGS and high HS-mGPS) had a significantly higher mortality risk than those with low risk (neither) (adjusted HR = 10.76; 95% CI: 5.38–21.54; *p* < 0.001).

**Table 2:** Association between each indicator and overall survival in colon cancer patients according to Cox regression models.

Variables	*N*	Model 1		Model 2		Model 3	
		HR (95%CI)	*p*-value	HR (95%CI)	*p*-value	HR (95%CI)	*p*-value
HGS							
As continues	811	0.98 (0.96–1.00)	0.029	0.98 (0.96–1.01)	0.158	0.98 (0.96–1.00)	0.097
High HGS	495	Ref.	–	Ref.	–	Ref.	–
Low HGS	316	3.72 (2.73–5.06)	<0.001	2.94 (2.12–4.07)	<0.001	3.20 (2.27–4.50)	<0.001
HS-mGPS							
0	413	Ref.	–	Ref.	–	Ref.	–
1	174	1.07 (0.73–1.55)	0.735	1.34 (0.91–1.98)	0.144	1.23 (0.82–1.85)	0.322
2	224	1.60 (1.70–2.19)	0.003	1.95 (1.37–2.78)	<0.001	1.88 (1.30–2.72)	0.001
Low HS-mGPS	413	Ref.	–	Ref.	–	Ref.	–
High HS-mGPS	398	1.36 (1.03–1.80)	0.032	1.64 (1.21–2.24)	0.002	1.55 (1.12–2.14)	0.008
HGS-HS-mGPS							
Low risk	221	Ref.	–	Ref.	–	Ref.	–
Median risk 1	192	8.93 (4.75–16.79)	<0.001	7.32 (3.84–13.95)	<0.001	8.66 (4.49–16.72)	<0.001
Median risk 2	274	4.00 (2.08–7.71)	<0.001	4.85 (2.47–9.50)	<0.001	5.06 (2.56–10.01)	<0.001
High risk	124	10.38 (5.45–19.75)	<0.001	10.09 (5.15–19.77)	<0.001	10.76 (5.38–21.54)	<0.001

Model 1: unadjusted. Model 2: adjusted for sex, age, BMI, aCCI, tumor T and N stage. Model 3: adjusted for Model 2 plus CEA, bowel obstruction, tumor location, operation type, CCI ≥ 26.2, tumor differentiation, and postoperative chemotherapy. HR, hazard ratio; CI, confidence interval.

**Figure 2:**
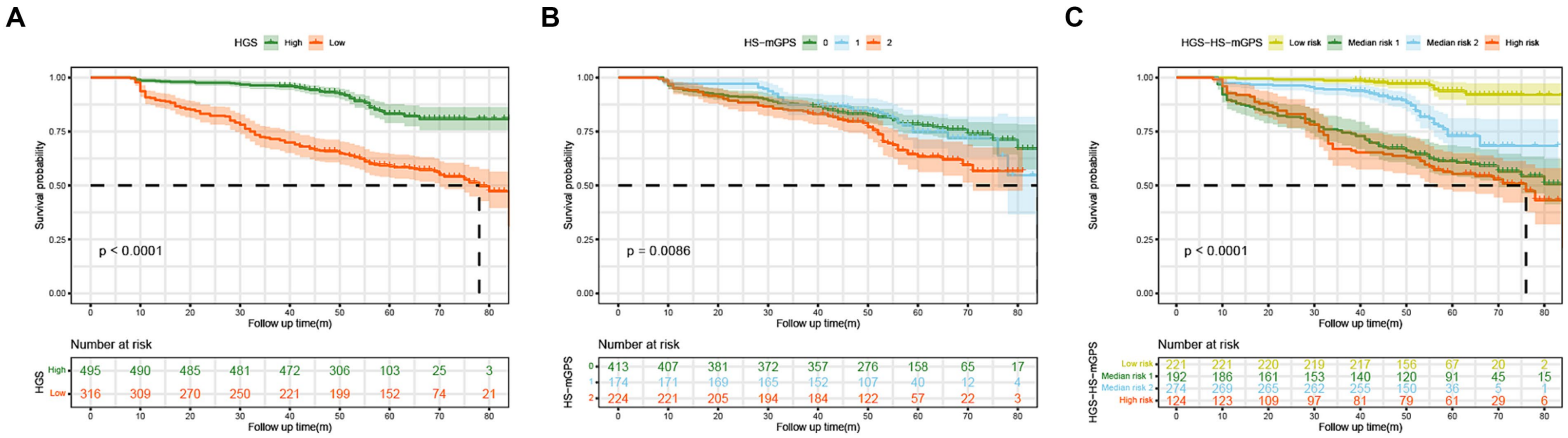
The Kaplan–Meier curves of colon cancer patients stratified by HGS **(A)**, HS-mGPS **(B)**, and HGS-HS-mGPS **(C)**. HGS, handgrip strength; HS-mGPS, high-sensitivity modified Glasgow prognostic score; HGS-HS-mGPS, the combination of HGS and HS-mGPS.

The Kaplan–Meier curves further analyzed the associations of different HGS, HS-mGPS, and risk groups with OS. Patients with low HGS had a worse OS than those with high HGS (*p* < 0.001) (Figure 2A). The high HS-mGPS was significantly associated with poor OS of colon cancer (*p* = 0.0086) (Figure 2B). Importantly, patients in the high-risk group had the worst OS than other three groups (*p* < 0.001) (Figure 2C), suggesting that the combination of low HGS and HS-mGPS could be a useful indicator of OS in patients with colon cancer.

### 3.3 Stratified analysis

Stratified analyses were performed to assess the association between HGS-HS-mGPS and OS in various subgroups (Table 3). Among colon cancer patients, the relationship between OS and HGS-HS-mGPS was not modified by sex, age, BMI, aCCI, bowel obstruction, CCI ≥ 26.2, TNM stage, and postoperative chemotherapy. Even in the younger subgroup (<60 years), the patients with low HGS and high HS-mGPS had a 10.13-fold higher death risk than those with high HGS and low HS-mGPS (adjusted HR = 10.13; 95% CI: 3.88–26.43; *p* < 0.001). There was no interaction between these factors and HGS-HS-mGPS (all *P* for interaction >0.05).

**Table 3:** HGS-HS-mGPS and colon cancer overall survival stratified by clinical and pathologic characteristics.

Stratification variables	Univariate analysis		Multivariate analysis		*p* for interaction
	HR (95%CI)	*p*-value	HR (95%CI)	*p*-value	
Sex					0.833
Male	15.67 (6.93–35.43)	<0.001	16.05 (6.57–38.13)	<0.001	
Female	8.96 (2.76–29.08)	<0.001	8.84 (2.66–29.41)	<0.001	
Age					0.071
<60 years	6.56 (2.71–15.87)	<0.001	10.13 (3.88–26.43)	<0.001	
≥60 years	11.89 (4.26–33.18)	<0.001	16.22 (5.42–48.53)	<0.001	
BMI (kg/m^2^)					0.587
<18.5	6.43 (0.67–61.80)	0.107	0.81 (0.05–12.74)	0.880	
18.5–24	12.89 (5.85–28.41)	<0.001	15.88 (6.94–36.35)	<0.001	
>24	3.89 (0.93–16.32)	0.063	6.33 (1.20–33.56)	0.030	
aCCI					0.550
0–1	10.56 (3.12–35.72)	<0.001	10.58 (2.89–38.76)	<0.001	
2–3	12.23 (3.44–43.43)	<0.001	16.67 (4.41–62.96)	<0.001	
≥4	7.68 (2.96–19.91)	<0.001	10.12 (3.57–28.73)	<0.001	
Bowel obstruction					0.577
No	8.33 (3.79–18.32)	<0.001	12.54 (5.40–29.10)	<0.001	
Yes	13.86 (4.24–45.28)	<0.001	16.55 (4.57–59.86)	<0.001	
CCI ≥ 26.2					0.077
No	12.25 (5.51–27.21)	<0.001	15.76 (6.68–37.19)	<0.001	
Yes	6.20 (2.07–18.58)	0.001	7.09 (2.24–22.45)	0.001	
TNM stage					0956
I	5.36 (0.54–53.15)	0.151	6.18 (0.52–73.25)	0.149	
II	8.53 (2.86–25.41)	<0.001	12.77 (3.73–43.73)	<0.001	
III	8.97 (3.80–21.16)	<0.001	12.72 (5.17–31.33)	<0.001	
Postoperative chemotherapy					0.090
No	10.98 (5.35–22.54)	<0.001	13.29 (6.09–28.99)	0.001	
Yes	13.95 (33.12–60.57)	<0.001	13.56 (2.98–61.63)	<0.001	

Multivariate Cox proportional models adjust for sex, age, BMI, aCCI, bowel obstruction, CCI ≥ 26.2, TNM stage, and postoperative chemotherapy, unless stratified by those variables. All HRs compare patients with low HGS and high HS-mGPS vs patients with high HGS and low HS-mGPS.

### 3.4 Nomogram construction and evaluation

**Figure 3:**
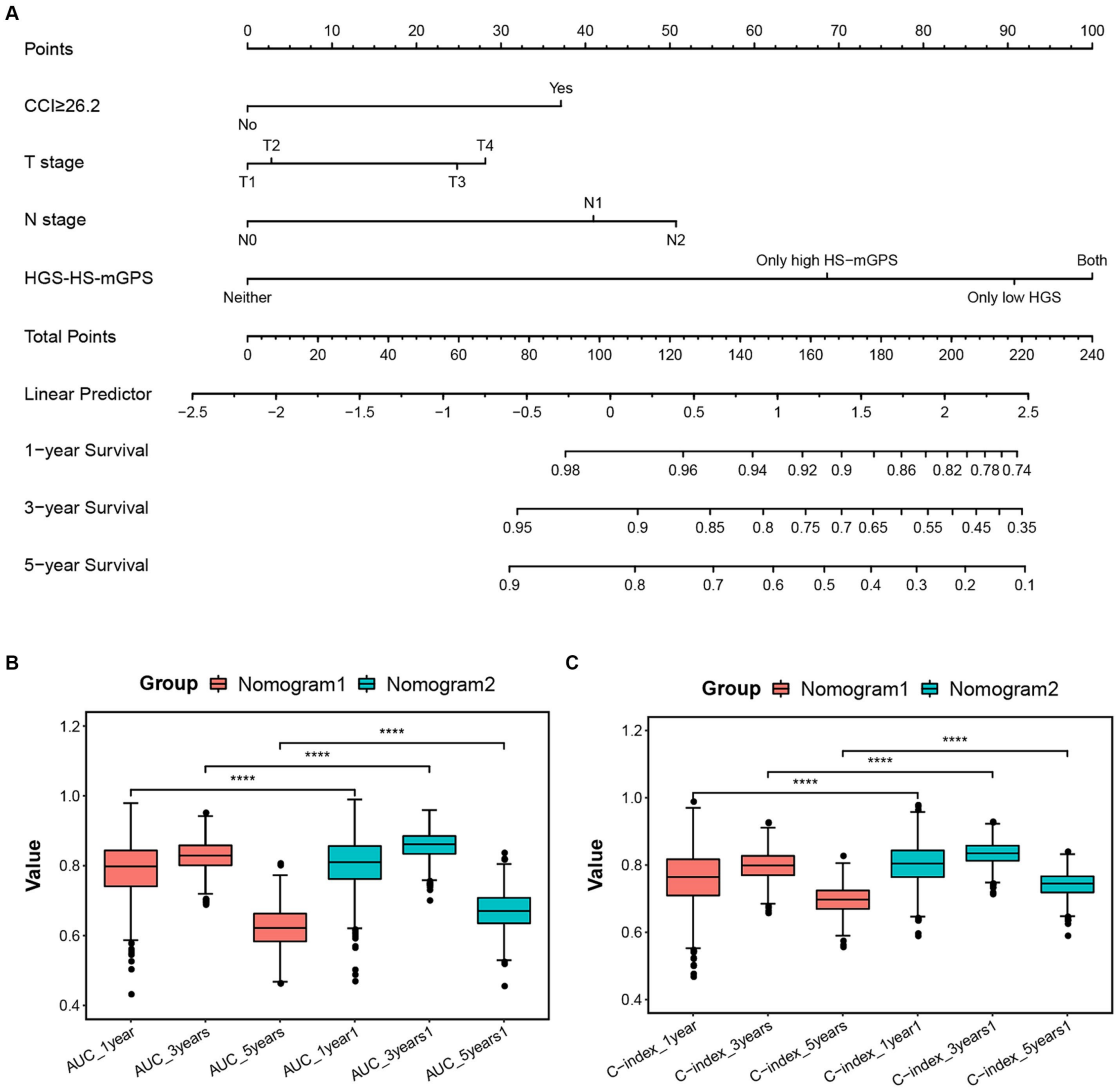
**(A)** Established nomogram for predicting 1-, 3-, and 5 year OS of colon cancer patients after radical resection by incorporating significant predictors (CCI ≥ 26.2, T stage, and N stage) and HGS-HS-mGPS. The AUC **(B)** and C-indices **(C)** of nomogram models for predicting OS at 1, 3, and 5 years postoperatively. Nomogram1: including only significant predictors; Nomogram2: including significant predictors and HGS-HS-mGPS. CCI, comprehensive complication index; HGS-HS-mGPS, the combination of HGS and HS-mGPS; AUC, area under the curve.

Univariate and multivariate analyses were performed to investigate the prognostic factors of colon cancer patients, the results are presented in Supplementary Table S2. Three prognostic variables, including CCI ≥ 26.2, advanced T stage, and N stage, were independently associated with OS. Based on these independent predictors, a nomogram predicting 1-, 3-, and 5 year OS probabilities was constructed (Supplementary Figure S2). In the nomogram, each indicator is allocated a score and then added to obtain the total scores that correspond to the estimated probability of OS. Of note, incorporating significant predictors and HGS-HS-mGPS into the model (nomogram2; Figure 3A) significantly increases the AUC and C-indices for predicting OS at 1, 3, and 5 years postoperatively (Figures 3B,C). The calibration plot of the nomogram 2 shows a good agreement between the predicted probability and actual observation (Supplementary Figures S3A–C). To evaluate the application value, the decision curve analyses indicated that nomogram 2 in predicting 1-, 3-, and 5 year OS was more beneficial than the model including significant predictors alone in a wide range of threshold probabilities (Supplementary Figures S3D–F).

### 3.5 Sensitivity analysis

We performed a sensitivity analysis in colon cancer patients with survival more than 12 months and the results were comparable to the main analysis (Table 4). The risk of death was significantly higher in high-risk groups than in low-risk groups (adjusted HR = 10.03; 95% CI: 4.94–20.37; *p* < 0.001), further demonstrating the prognostic value of HGS-HS-mGPS for colon cancer.

**Table 4:** Sensitivity analyses of colon cancer patients surviving more than 12 months.

Variables	*N*	Model 1		Model 2		Model 3	
		HR (95%CI)	*p*-value	HR (95%CI)	*p*-value	HR (95%CI)	*p*-value
HGS							
As continues	774	0.99 (0.97–1.01)	0.295	0.99 (0.97–1.02)	0.465	0.98 (0.96–1.01)	0.219
High HGS	488	Ref.	–	Ref.	–	Ref.	–
Low HGS	286	3.29 (2.35–4.59)	<0.001	2.77 (1.94–3.95)	<0.001	3.17 (2.20–4.57)	<0.001
HS-mGPS							
0	392	Ref.	–	Ref.	–	Ref.	–
1	169	1.22 (0.81–1.85)	0.337	1.39 (0.91–2.12)	0.131	1.32 (0.84–2.06)	0.227
2	213	1.80 (1.27–2.55)	0.001	2.06 (1.39–3.05)	<0.001	2.07 (1.38–3.11)	<0.001
Low HS-mGPS	392	Ref.	–	Ref.	–	Ref.	–
High HS-mGPS	382	1.54 (1.13–2.10)	0.007	1.70 (1.21–2.39)	0.002	1.68 (1.72–2.40)	0.005
HGS-HS-mGPS							
Low risk	221	Ref.	–	Ref.	–	Ref.	–
Median risk 1	171	6.60 (3.46–12.59)	<0.001	5.30 (2.72–10.28)	<0.001	6.80 (3.44–13.43)	<0.001
Median risk 2	267	3.51 (1.81–6.84)	<0.001	3.61 (1.83–7.15)	<0.001	4.06 (2.03–8.14)	<0.001
High risk	115	8.88 (4.62–17.05)	<0.001	8.52 (4.30–16.87)	<0.001	10.03 (4.94–20.37)	<0.001

Model 1: unadjusted. Model 2: adjusted for sex, age, BMI, aCCI, tumor T and N stage. Model 3: adjusted for Model 2 plus CEA, bowel obstruction, tumor location, operation type, CCI ≥ 26.2, tumor differentiation, and postoperative chemotherapy.

## 4 Discussion

To our knowledge, this study is the first to establish sex-specific cut-off points of HGS for colon cancer patients and explore the prognosis values of HGS, HS-mGPS, and HGS-HS-mGPS for OS. We discovered that low HGS and high HS-mGPS were independent prognostic factors for patients with colon cancer, and their combination significantly correlated with poor survival. The nomogram, incorporating HGS-HS-mGPS and clinicopathological factors, could effectively predict the OS of colon cancer with the good performance of discrimination and calibration.

Handgrip strength is a common parameter for assessing the muscle strength and nutritional status (22). Several previous studies indicated that HGS was a robust predictor of morbidity and mortality in peritoneal dialysis, respiratory disease, cardiovascular disease, and patients with cancer (13, 14, 23, 24). However, the sex-specific HGS cut-off points in colon cancer patients and the association between HGS and postoperative OS of colon cancer remained unclear. In this study, we calculated the HGS cut-off values as <28.8 kg for males and <19.72 kg for females. These values were higher than the cut-off values for defining sarcopenia in Asian Working Group for Sarcopenia (AWGS) guideline (25) and patients with pan-cancer in other studies (10, 14). This discrepancy is reasonable and may be attributed to differences in cancer types among the population. Consistent with previous studies, low HGS was significantly associated with poor OS of patients with colon cancer. Nevertheless, HGS only reflects muscle phenotype and is easily affected by other factors during measurement. The diagnosis of malnutrition requires at least one phenotypic criterion and one etiological criterion (26). Therefore, the inflammatory indicator was included in the present study.

The HS-mGPS, ranging from 0 to 2, is a potential biomarker produced by the combination of two laboratory indices (CRP and albumin). Accumulating evidence suggested that the HS-mGPS had independent prognostic value for different types and stages of cancer, including gastric, hepatocellular, and oropharyngeal cancer (27–29). Likewise, a HS-mGPS of 2 in cancer patients acted as an unfavourable prognostic indicator for disease-free survival and disease-specific survival (30, 31). Consistently, the present study demonstrated a significant association between high HS-mGPS and poor OS of patients with colon cancer. In addition, the prognostic value of the HS-mGPS was reported to surpass that of neutrophil-to-lymphocyte ratio, platelet-to-lymphocyte ratio, and conventional mGPS in certain cancers (17, 18, 32, 33). Therefore, including the HS-mGPS into the preoperative work-up may help identify colon cancer patients with a high risk of mortality.

Surprisingly, the combined effect of HGS and HS-mGPS on the postoperative survival of patients with colon cancer was investigated in our study. The HGS-HS-mGPS classification included the low-risk, median-risk, and high-risk groups. As expected, the patients in the high-risk group had the greatest risk of death than other groups. Furthermore, including the HGS-HS-mGPS into the prediction model significantly improved performance in predicting OS probabilities. The mechanism underlying this finding may be attributed to the synergistic effect of muscle wasting and high inflammatory load. Skeletal muscle is the largest metabolic organ, accounting for approximately 80% of insulin-stimulated glucose uptake (34). Muscle loss results in insulin resistance and increased activity of IGF-1, which can promote colorectal tumour progression (35). Regardless of the stage of the cancer, low muscle mass negatively impacts surgical complications, physical function, and quality of life, ultimately reducing the possibility of survival (36). Muscle wasting also reduces the secretion of anti-inflammatory factors, such as IL-6, IL-8, and IL-15, leading to the excessive inflammatory burden, which in turn exacerbates the loss of muscle mass (15, 37). Moreover, proinflammatory mediators in serum have been demonstrated to reduce muscle strength (38). Our study showed a significant association between low HGS and high CRP levels, which is consistent with previous studies (38, 39). CRP is possibly an active mediator of cancer progression and aggressive phenotype, rather than merely a passive reflection of the inflammatory process. CRP could alter the expression of proto-oncogenes and inhibitory genes through different pathways, facilitating cancer cell proliferation, migration, invasion, and chemotherapy resistance (40). Therefore, muscle phenotype and inflammation interact and the combination of HGS and HS-mGPS exhibits a significant prognostic value for patients with colon cancer. In addition, HGS and HS-mGPS are easy and low-cost examinations to incorporate into clinical practice.

Patients with colon cancer are susceptible to malnutrition, which could significantly influence their mortality risk (41). Poor nutrition further aggravates muscle loss and activates the inflammation cascade. A recent review indicated that in colorectal cancer patients at high nutritional risk, the chance of progressing to cachexia is up to 31.8% (42). Proper physical exercise is an effective therapeutic intervention to maintain muscle mass and function, which offsets the morbidity and mortality of cancer cachexia (43). In addition, individualized nutritional support during the hospital stay could reduce mortality and improve the quality of life in cancer patients with nutritional risk (44). However, only 33.9% of clinicians follow the recommendations in clinical nutrition guidelines for cancers (45). Hence, more attention should be devoted to the preoperative nutritional status of colon cancer patients with low HGS or elevated inflammatory markers.

Of note, our study has some limitations. First, this was a single-center study with a small sample size, which limits the generalizability of the results. Multiple-center studies with wider geographic recruitment are necessary to validate the findings of the present study. Second, some unmeasured confounders, such as anorexia and nutrition risk screening, could have effects on the results of our analyses. Third, only perioperative data were collected, which may not reflect the latest advances in colon cancer treatment, such as immunotherapy, possibly leading to an underestimation of survival. Finally, the nomogram constructed in this study needs further external validation.

In conclusion, we established the sex-specific HGS cut-off values for patients with colon cancer. Low HGS and high HS-mGPS were adversely associated with colon cancer prognosis. Furthermore, low HGS had combined effects with high HS-mGPS on the poor survival of patients with colon cancer. The established nomogram model can effectively predict the long-term survival in colon cancer patients following radical resection. These findings indicate the usefulness of HGS and HS-mGPS measurements in clinical practice for improving patient assessment, cancer prognosis, and precise intervention.

## Data availability statement

The raw data supporting the conclusions of this article will be made available by the authors, without undue reservation.

## Ethics statement

The studies involving humans were approved by the ethics committee of the Affiliated Hospital of Jiangnan University (No. 20230046). The studies were conducted in accordance with the local legislation and institutional requirements. Written informed consent for participation was not required from the participants or the participants’ legal guardians/next of kin in accordance with the national legislation and institutional requirements.

## Author contributions

YiS: Writing – review & editing, Writing – original draft, Supervision, Funding acquisition, Conceptualization. YuS: Writing – review & editing, Writing – original draft, Formal analysis, Data curation. XS: Writing – review & editing, Visualization, Data curation. ZY: Writing – review & editing, Formal analysis, Data curation. BX: Writing – review & editing, Supervision, Formal analysis. CB: Writing – review & editing, Conceptualization.

## Footnotes

^1^ www.assessurgery.com
