# Supplementary material for: Combination of handgrip strength and high-sensitivity modified Glasgow prognostic score predicts survival outcomes in patients with colon cancer
Source: Front Nutr. 2024 Jul 1;11:1421560. doi: 10.3389/fnut.2024.1421560 (PMC11247022; doi:10.3389/fnut.2024.1421560)
Supplement: Supplementary file 1 [file Data_Sheet_1.PDF]

Supplementary Material

1 Supplementary Figures

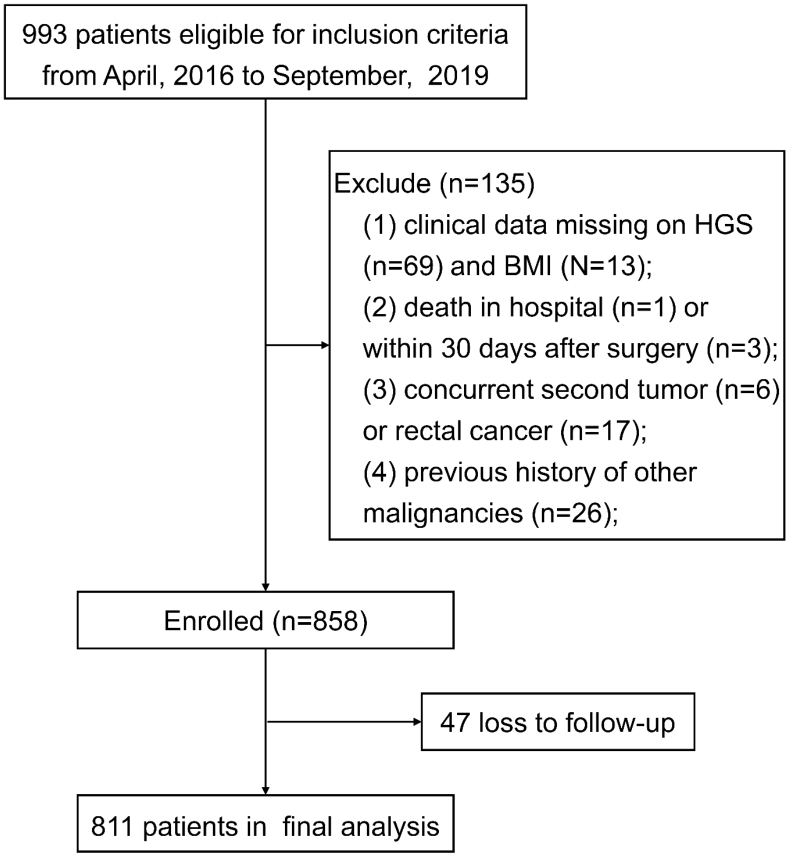

Figure S1. Flow chart of enrolled patients.

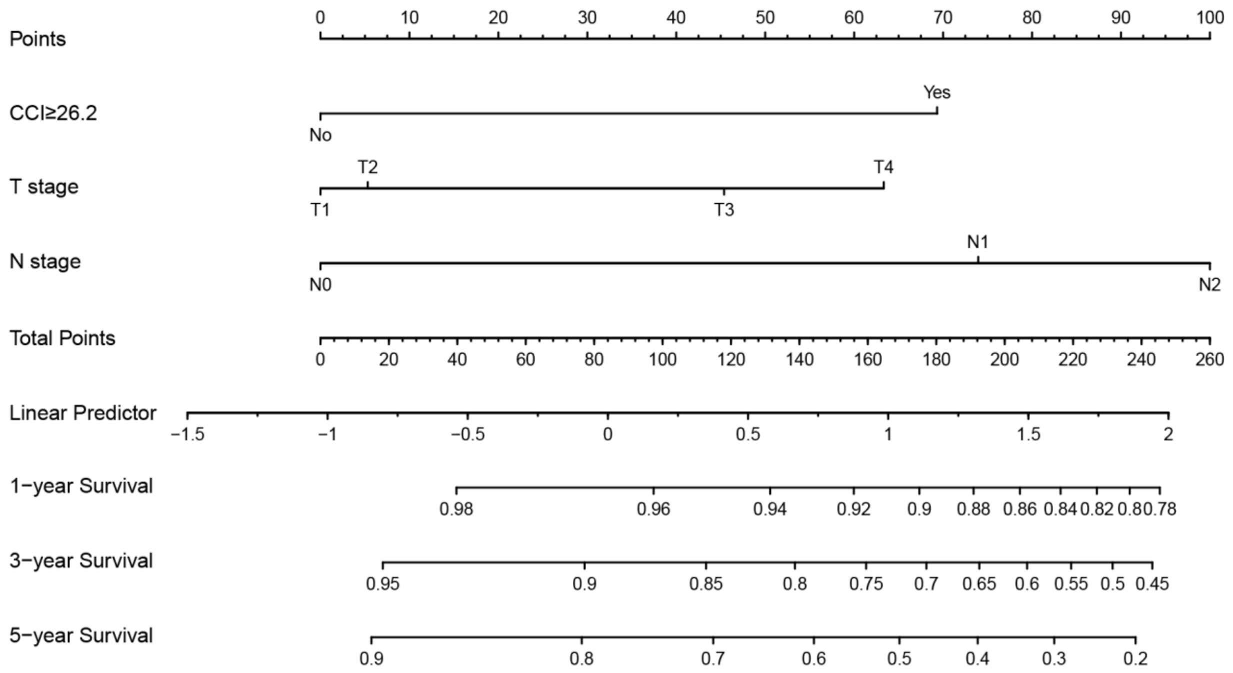

Figure S2. Established nomogram for predicting 1-, 3-, and 5-year OS of colon cancer patients after radical resection by incorporating significant predictors (CCI ≥ 26.2, T stage, and N stage).

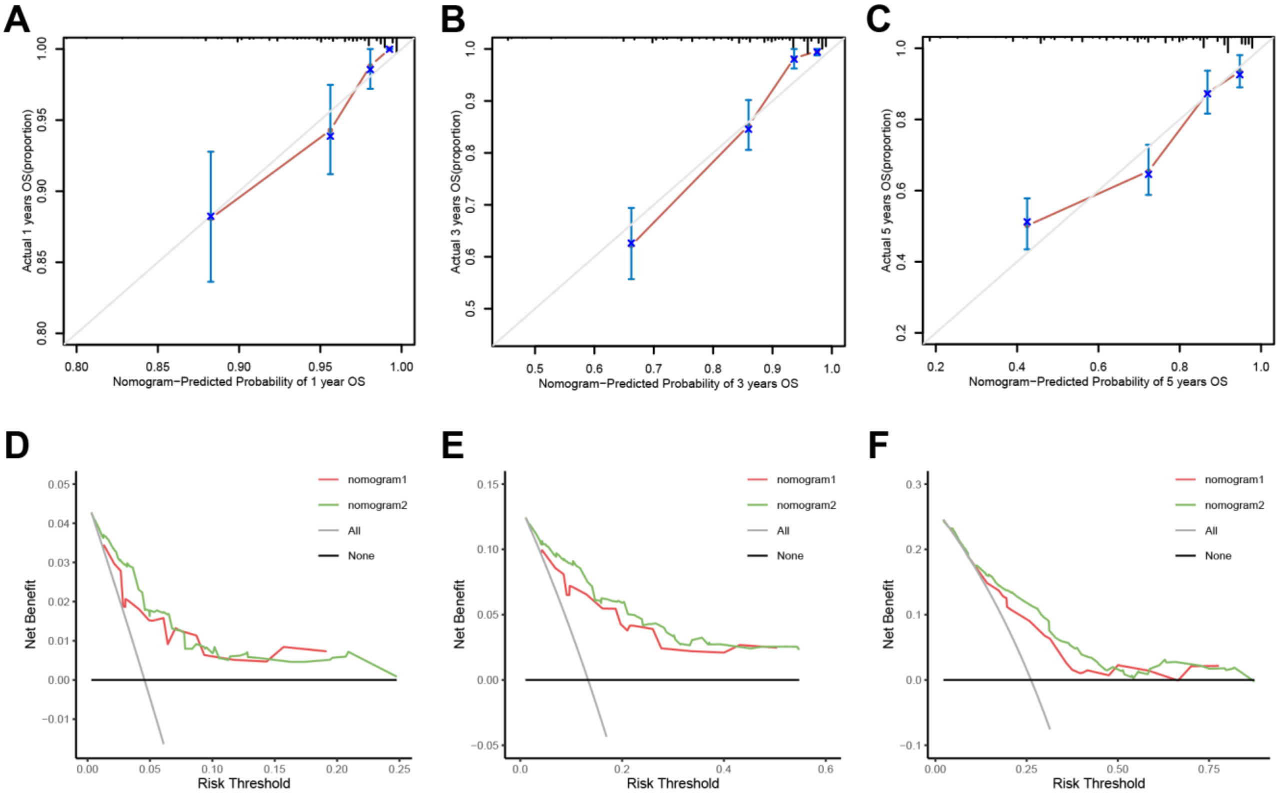

Figure S3. Calibration curve of the nomogram for predicting 1-, 3-, and 5-year OS of colon cancer patients after radical resection **(A, B, C)**. The decision curves of nomogram models to predict 1-, 3-, and 5-year OS **(D, E, F)**. Red line: nomogram1. Green line: nomogram2. Grey line: all patients died. Black line: none of the patients died. Nomogram1: including only significant predictors; Nomogram2: including significant predictors and HGS-HS-mGPS. HGS-HS-mGPS, the combination of HGS and HS-mGPS.

2 Supplementary Tables

Table S1. Baseline characteristics of patients with colon cancer according to different HGS and HS-mGPS levels.

Variables	HGS			HS-mGPS		
	High HGS(n = 495)	Low HGS(n = 316)	*P*-Value	Low HS-mGPS(n = 413)	High HS-mGPS(n = 398)	*P*-Value
Sex, *n* (%)						
Male	272(45.1)	174(44.9)	0.975	292(70.7)	154(38.7)	< 0.001
Female	223(54.9)	142(55.1)		121(29.3)	244(61.3)	
Age, *n* (%)						
< 60 years	284(57.4)	126(39.9)	< 0.001	227(55.0)	183(46.0)	0.011
≥ 60 years	211(42.6)	190(60.1)		186(45.0)	215(54.0)	
BMI (kg/m^2), *n* (%)						
< 18.5	31(6.3)	32(10.1)	0.002	39(9.4)	24(6.0)	0.192
18.5-24	375(75.8)	252(79.7)		314(76.0)	313(78.6)	
> 24	89(18.0)	32(10.1)		60(14.5)	61(15.3)	
HGS, kg, mean (SD)	29.64(5.28)	19.44(6.26)	< 0.001	25.99(7.06)	25.33(8.02)	0.210
aCCI, *n* (%)						
0-1	100(20.2)	72(22.8)	0.395	85(20.6)	87(21.9)	0.011
2-3	203(41.0)	115(36.4)		182(44.1)	136(34.2)	
≥ 4	192(38.8)	129(40.8)		146(35.4)	175(44.0)	
CEA(μg/L), n (%)						
< 5	311(62.8)	181(57.3)	0.115	258(62.5)	234(58.8)	0.284
≥ 5	184(37.2)	135(42.7)		155(37.2)	146(41.2)	
Albumin (g/L), mean (SD)	39.23(6.01)	36.01(5.12)	< 0.001	40.23(3.68)	34.25(6.11)	< 0.001
CRP (mg/L), median (interquartile range)	1.10(0.90, 7.81)	2.20(2.35, 26.25)	< 0.001	1.31(1.35, 2.12)	10.21(1.25, 24.48)	< 0.001
Bowel obstruction, n (%)						
No	361(72.9)	224(70.9)	0.527	318(77.0)	267(67.1)	0.002
Yes	134(27.1)	92(29.1)		95(23.0)	131(32.9)	

Tumor location, n (%)						
Right-sided	198(40.0)	113(35.8)	0.395	1453(35.1)	166(41.7)	0.127
Transverse	34(6.9)	27(8.5)		35(8.5)	26(6.5)	
Left-sided	263(53.1)	176(56.7)		233(56.4)	206(51.8)	
Operation type, n (%)						
Laparoscopy	413(83.4)	248(78.5)	0.076	339(82.1)	322(80.9)	0.666
Laparotomy	82(16.6)	68(21.5)		74(17.9)	76(19.1)	
CCI ≥ 26.2, n (%)						
No	421(85.1)	239(75.6)	0.001	329(79.7)	331(83.2)	0.200
Yes	74(14.9)	77(24.4)		84(20.3)	67(16.8)	
Differentiation, n (%)						
High	41(8.3)	22(7.0)	0.233	33(8.0)	30(7.5)	0.128
Moderate	199(40.2)	146(46.2)		189(45.8)	156(39.2)	
Low	255(51.5)	148(46.8)		191(46.2)	212(53.3)	
T stage, n (%)						
T1	112(22.6)	29(9.2)	< 0.001	57(13.8)	84(21.1)	0.009
T2	142(28.7)	44(13.9)		91(22.0)	95(23.9)	
T3	132(26.7)	58(18.4)		96(23.2)	94(23.6)	
T4	109(22.0)	185(58.5)		169(40.9)	125(31.4)	
N stage, n (%)						
N0	312(63.0)	141(44.6)	< 0.001	224(54.2)	229(57.5)	0.017
N1	149(30.1)	86(27.2)		112(27.1)	123(30.9)	
N2	34(6.9)	89(28.2)		77(18.6)	46(11.6)	
TNM stage, n (%)						
I	174(35.2)	37(11.7)	< 0.001	89(21.5)	122(30.7)	0.008
II	142(28.7)	108(34.2)		141(34.1)	109(27.4)	
III	179(36.2)	171(54.1)		183(44.3)	167(42.0)	
Postoperative chemotherapy, n (%)						
No	210(42.4)	161(50.9)	0.017	203(49.2)	168(42.2)	0.047
Yes	285(57.6)	155(49.1)		210(50.8)	230(57.8)	

Continuous variables were presented as means (standard deviations, SD) or median (interquartile range). Categorical variables were expressed as frequencies (percentages). HGS, hand grip strength; HS-mGPS, high-sensitivity modified Glasgow Prognostic Score; BMI, body mass index; aCCI, age-adjusted Charlson Comorbidity Index; CEA, carcinoembryonic antigen; CRP, C-reactive protein; CCI, comprehensive complication index. Differences in baseline characteristics were compared using the chi-square test, t-test or mann–whitney U-test.

Table S2. Univariate and multivariate Cox regression analysis of OS in patients with colon cancer.

Variables	Univariate analysis		Multivariate analysis	
	HR (95% CI)	*P*-Value	HR (95% CI)	*P*-Value
Sex				
Male vs. Female	1.07(0.81-1.42)	0.634	-	-
Age (year)				
≥ 60 vs. < 60	1.26(0.95-1.66)	0.106	-	-
BMI (kg/m^2)				
18.5-24 vs.< 18.5	1.13(0.67-1.93)	0.643	-	-
> 24 vs.< 18.5	0.88(0.47-1.68)	0.707	-	-
aCCI				
2-3 vs. 0-1	0.80(0.56-1.16)	0.237	-	-
≥ 4 vs. 0-1	0.87(0.61-1.24)	0.440	-	-
CEA(μg/L)				
≥ 5 vs.< 5	1.21(0.92-1.60)	0.179	-	-
Bowel obstruction				
Yes vs. No	1.00(0.74-1.37)	0.983	-	-
Tumor location				
Transverse vs. Right-sided	1.34(0.82-2.19)	0.240	-	-
Left-sided vs. Right-sided	0.90(0.67-1.21)	0.501	-	-
Operation type				
Laparoscopy vs. Laparotomy	0.91(0.65-1.29)	0.600	-	-
CCI ≥ 26.2				
Yes vs. No	2.27(1.68-3.07)	<0.001	2.33(1.71-3.17)	<0.001
Differentiation				
Moderate vs. High	0.65(0.39-1.08)	0.098	-	-
Low vs. High	1.03(0.63-1.67)	0.921	-	-
T stage				
T2 vs. T1	1.12(0.62-2.02)	0.713	0.92(0.51-1.67)	0.785
T3 vs. T1	1.76(1.01-3.05)	0.045	1.55(0.88-2.72)	0.129
T4 vs. T1	2.81(1.70-4.64)	<0.001	1.91(1.13-3.24)	0.016
N stage				
N1 vs. N0	2.56(1.83-3.59)	<0.001	2.08(1.33-3.25)	0.001
N2 vs. N0	4.08(2.85-5.83)	<0.001	2.84(1.78-4.45)	<0.001
Postoperative chemotherapy				
Yes vs. No	0.36(0.27-3.73)	<0.001	0.78(0.51-1.20)	0.257

HR, hazard ratio; CI, confidence interval.
